# The proportion, origin and pro-inflammation roles of low density neutrophils in SFTS disease

**DOI:** 10.1186/s12879-019-3701-4

**Published:** 2019-02-04

**Authors:** Yajiao Li, Huiyu Li, Hua Wang, Hong Pan, Huixia Zhao, Honglin Jin, Shenghua Jie

**Affiliations:** 1 Union Hospital, Tongji Medical College, Huazhong University of Science and Technology, Wuhan, 430022 China; 2 Department of Infectious Diseases, Union Hospital, Tongji Medical College, Huazhong University of Science and Technology, Wuhan, 430022 China; 3 Kingstar Global, Wuhan, 430022 China; 4 Cancer Center, Union Hospital, Tongji Medical College, Huazhong University of Science and Technology, Wuhan, 430022 China

**Keywords:** LDNs, Conversion, NDNs, SFTS, SFTSV

## Abstract

**Background:**

Severe fever with thrombocytopenia syndrome (SFTS) is a novel emerging viral infectious disease. We explored the percentage, origins and functional roles of low density neutrophils (LDNs), one of the neutrophils subsets, in SFTS.

**Methods:**

The LDNs and normal density neutrophils (NDNs) from blood of SFTS and normal volunteers which were collected separately. The percentage, origins and the phagocytic capability of SFTS viral (SFTSV) of LDNs were investigated by flow cytometry and real time PCR. The capacity of LDNs to secrete cytokines and to damage endothelial cells was assessed by ELISA and flow cytometry.

**Results:**

We observed that the proportion of LDNs increased dramatically compared with the healthy donors and became the dominant circulating neutrophil population in SFTS patients. Interestingly, the NDNs from the normal donors could switch to LDNs under the SFTS environment. Moreover, SFTSV load in LDNs was significantly higher than that of NDNs in the severe SFTS patients. In addition, the LDNs secreted much higher levels of pro-inflammatory cytokines than NDNs in SFTS and could induce endothelial cell injury.

**Conclusion:**

The NDNs can be converted to LDNs. This conversion mechanism could become the source of LDNs. The LDNs in severe SFTS patient could engulf more SFTSV and exhibit pro-inflammation functions.

**Trial registration:**

The Ethics Committee of Tongji Medical College, Huazhong University of Science and Technology (IORG No: IORG0003571) gave a final APPROVAL for the study.

## Background

Severe fever with thrombocytopenia syndrome (SFTS) is a novel infectious disease caused by SFTS virus (SFTSV), a new type of bunyavirus, which is a phlebovirus in the Bunyaviridae family [1–3]. SFTSV undergoes rapid changes owing to evolutions, gene mutations, and reassortments between different strains of SFTSV. From 2010 to 2017, SFTS cases were reported in 23 provinces of China and the numbers are increasing each year [4]. SFTS is often characterized by fever, thrombocytopenia, leukocytopenia and gastrointestinal symptoms. The clinical symptoms of SFTS patients usually become exacerbated quickly to the multiple organ dysfunction syndromes (MODS). Due to this reason, the mortality rate of SFTS reached a high number of 30%, with higher risks to elder people [1]. SFTS greatly threatened public health. Further explorations SFTS pathological mechanisms are urgently needed.

The number of neutrophils was abnormally decreased in SFTS patients [5–7]. Although the number of reported SFTS has increased in the recent years, the neutrophils remains one of the less studied in SFTS. It is well known that neutrophils are the first immune cell population recruited to sites of infection [8] and their functions in virus infection was controversial because neutrophils exhibit both protective and pathological functions [8]. Accumulating evidence support the existence of distinct neutrophil subsets in circulating blood in density gradient preparations and this population of neutrophils is termed LDNs and NDNs [9–12].Circulating LDNs are seldomly detected in healthy controls, however, LDNs are abundant in mycobacterial infections and are associated with the severity of tuberculosis [11].More importantly, the number of LDNs were increased in the circulating blood of patients with human immunodeficiency virus [13]. It has reported that LDNs display impaired neutrophil function and immunosuppressive properties, characteristics that are in stark contrast to those of NDNs. Recently, we have demonstrated the existence of abnormal percentage of LDNs in the circulation blood of SFTS patients [14]. However, the source of LDNs in SFTS remains unclear. Therefore, this study was to explore the origin and functional roles of LDNs in SFTS, and may explain pathogenesis of SFTS disease.

## Methods

### Patients

Forty SFTS patients and 40 volunteers were recruited from the Department of Infectious Diseases of Union Hospital, Tongji Medical College, Huazhong University of Science and Technology between May 2015 and July 2017. The procedures were in accordance with the ethical standards of the Helsinki Declaration. Subjects with the SFTS were diagnosed according to clinical symptom and laboratory examination.

The median interval of the SFTS patients between the onset of illness and extraction of peripheral blood at admission was 3 days (range, 1–5 days). There were 25 women and 15 men. The median age of the patients was 59.50 ± 8.85 years (range, 40–72 years). The healthy volunteers without any diseases, were recruited from April 2015 to July 2017. The volunteers’ health conditions were verified via health examination, and the volunteers were subjected to no treatments with drugs prior to and during the investigation phase. There were 20 women and 20 men. The median age of normal controls was 54.70 ± 7.82 years old (range, 40–66 years). There were no significant differences in the average age and sex ratio between SFTS patients and controls.

### Blood samples

Collection of blood from SFTS patients and healthy volunteers was approved by Tongji Medical College, Huazhong University of Science and Technology institutional review board. Peripheral blood from SFTS patients or the healthy volunteer blood was drawn by venipuncture and collected using EDTA containers. Blood samples were transferred to the lab for analysis for different experiments.

### Density separation

Peripheral blood was diluted with phosphate buffered saline (PBS) and was centrifuged on a cushion of Ficoll-Histopaque in a centrifuge tube for 30 min at 400×g at room temperature. LDNs were collected from the PBMCs interface, diluted with PBS and centrifuged for 5 min at 300×g. Red blood cells were eliminated by hypotonic lysis. The collected LDNs were washed twice with PBS. NDNs were collected from the granulocyte-erythrocyte pellet by brief hypotonic lysis and were washed twice with PBS. The cells were suspended respectively in RPMI-1640 for analyzing immediately by flow cytometry or other experiments. LDNs and NDNs were identified as CD15+, CD45 + .

### Isolation of LDNs and NDNs

LDNs and NDNs were purified and performed on a FACS Vantage cell separator. Under sterile conditions, tube was incubated with APC-labeled CD15 +, PerCP-labeled CD45+ monoclonal antibody, then 488 mm laser activated fluorescence to separate cells, and 2% FBS in PBS as sheath fluid.

### Adoptive transfers

The isolated NDNs from health volunteers were incubated for 4 h, 12 h, 24 h, 48 h in RPMI-1640 medium supplemented with 100 μl plasma of SFTS patient. The cells were harvested at different time points and isolated LDNs and NDNs using Histopaque 1077 and centrifuged for 30 min. The LDNs and NDNs were suspended in RPMI-1640 for analyzing by flow cytometry.

### Measurements of SFTSV RNA in LDN, NDNs and serum from SFTS

Next, we tested the SFTSV load in LDNs and NDNs from SFTS patients and the SFTS serum. The Blood RNA Kit (Omega) were used to extract SFTSV RNA from serum, LDNs and NDNs of SFTS according to the manufacturer’s instruction. The SFTSV RNA in serum, LDNs and NDNs were detected by a commercially available quantitative real-time PCR diagnostic kit (SFDA, China), according to the manufacturer’s instructions.

### Cytokine quantifications

We also detected inflammatory cytokines concentration from the serum of SFTS and normal controls, the cultured supernatant of LDNs and NDNs from SFTS. The LDNs, NDNs from SFTS were cultured in RPMI-1640/1% FBS for 48 h in the presence of SFTSV (3260 copies/ml).Supernatants were then harvested. The concentration of the cytokines IL-2, IL-4, IL-6, IL-8, IL-10, IL-17, TNF-α and IFN-γ were quantified using Human Cytometric Bead Array (CBA) Kit (BD BioScience) as described by the manufacturer’s instructions.

### The endothelial cell cytotoxicity assay

The HUVECs were purchased from ATCCTM Cell Biology, USA and were cultured in RPMI-1640 medium supplemented with 10% FBS. The cells were grown in 5% CO_2_ at 37 °C. HUVECs were plated into a 6-well plate at a density of 2 × 10^5^/well in RPMI-1640 supplemented with 10% FBS. Transwell (Corning, USA) inserts with an 0.4 μm pore size were inserted into the 6-well plates, while 900 μL (2 × 10^5^cells) of activated and unactivated LDNs were, added respectively to the upper wells in RPMI-1640 supplemented with 10% FBS. These cells were then co-cultured in 5% CO_2_at 37 °C for 24 h or 48 h. After incubation, the HUVECs were trypsinized. Following the instructions of Annexin V-FITC apoptosis detection kit (BD Pharmingen TM, USA), apoptosis rate of HUVECs was detected by flow cytometry.

### The ethics statement

All participants signed informed consents before participation. The research protocol was approved by the Ethics Committee of Tongji Medical College, Huazhong University of Science and Technology.

### Statistical analyses

Unless indicated otherwise, results were summarized as means and Standard Error of Mean (mean ± SEM). Results were analyzed using the statistical software package SPSS 18.0 (SPSS, an IBM Company, Armonk, NY, USA). Comparison of various groups was performed using Student’s t tests when the data were normally distributed; otherwise, the Mann-Whitney test was used. The correlation analysis of 2 variables among virus load, LDNs, NDNs, and cytokines were calculated using Pearson and Spearman test. *P* < 0.05 was considered statistically significant. The statistical graphs were performed by Graph Pad Prism 5.00 (GraphPad Software, San Diego, CA, USA).

## Results

### The existence of abnormally increased proportion of LDNs in SFTS patients

**Fig. 1:**
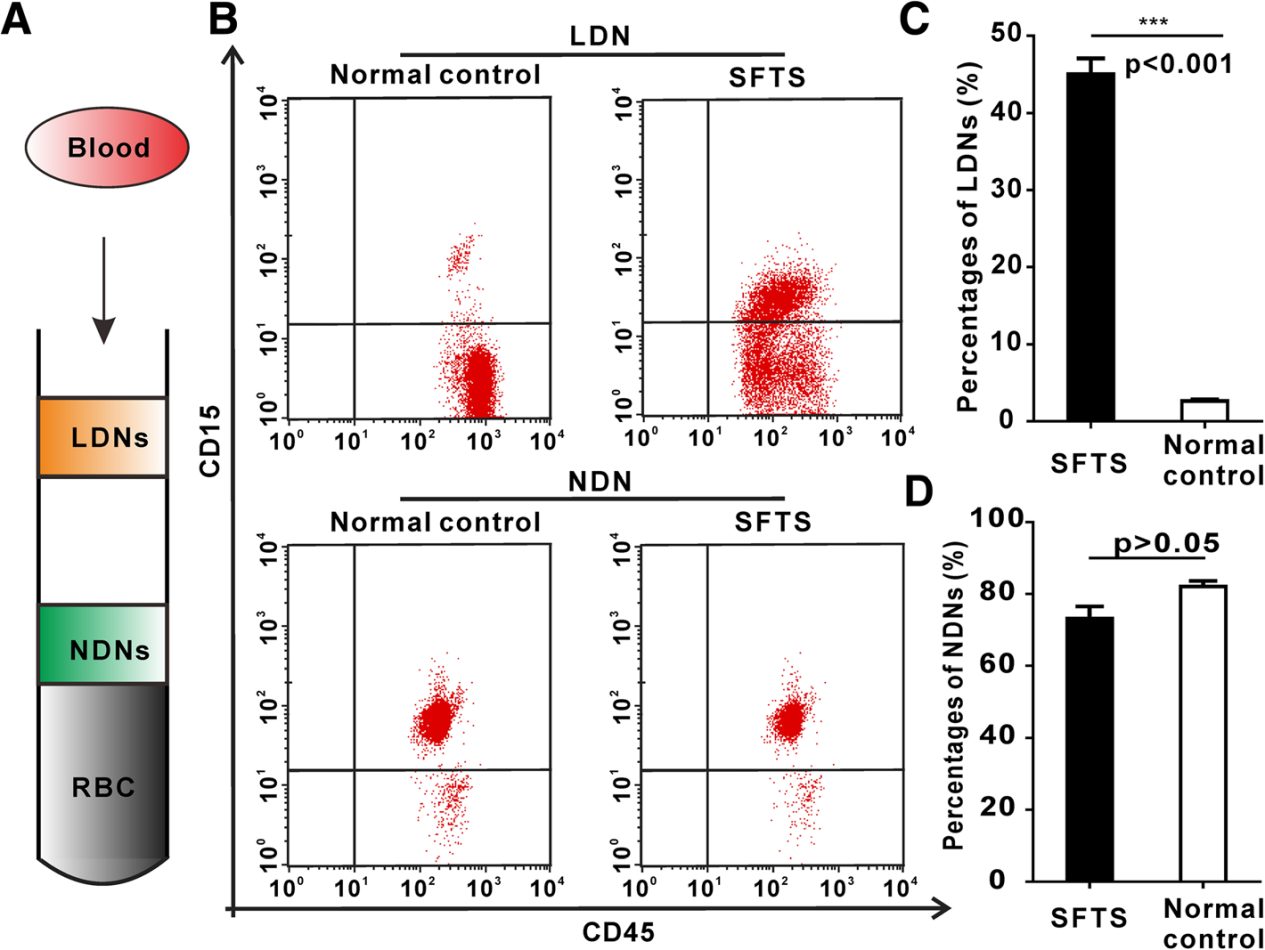
Enrichment of SFTS LDNs. **a** Separation of whole blood of SFTS on a Histopaque density separating LDNs, NDNs and RBCs. **b** Results show representative dot plots of LDNs. **c** and **d** Percentages of LDNs and NDNs in peripheral blood mononuclear cells (PBMCs). Error bars represent ±SEM

We comfirmed that there were two neutrophil subsets LDNs and NDNs present in circulating blood of SFTS patients (Fig. 1a). The proportion of LDNs was significantly increased in SFTS patients (46.8 ± 14.1%), compared to that of normal controls (2.6 ± 1.4)% (*p* < 0.001) (Fig. 1b, c). The percentages of NDNs from the SFTS patients was diminished (73.10 ± 1.57)%, compared to that of normal controls (82.06 ± 3.38)% (*p* > 0.05). There were no statistical significances between SFTS and normal control (*p* > 0.05) (Fig. 1b, d).

### NDNs can be reversed to LDNs in the SFTS environment

**Fig. 2:**
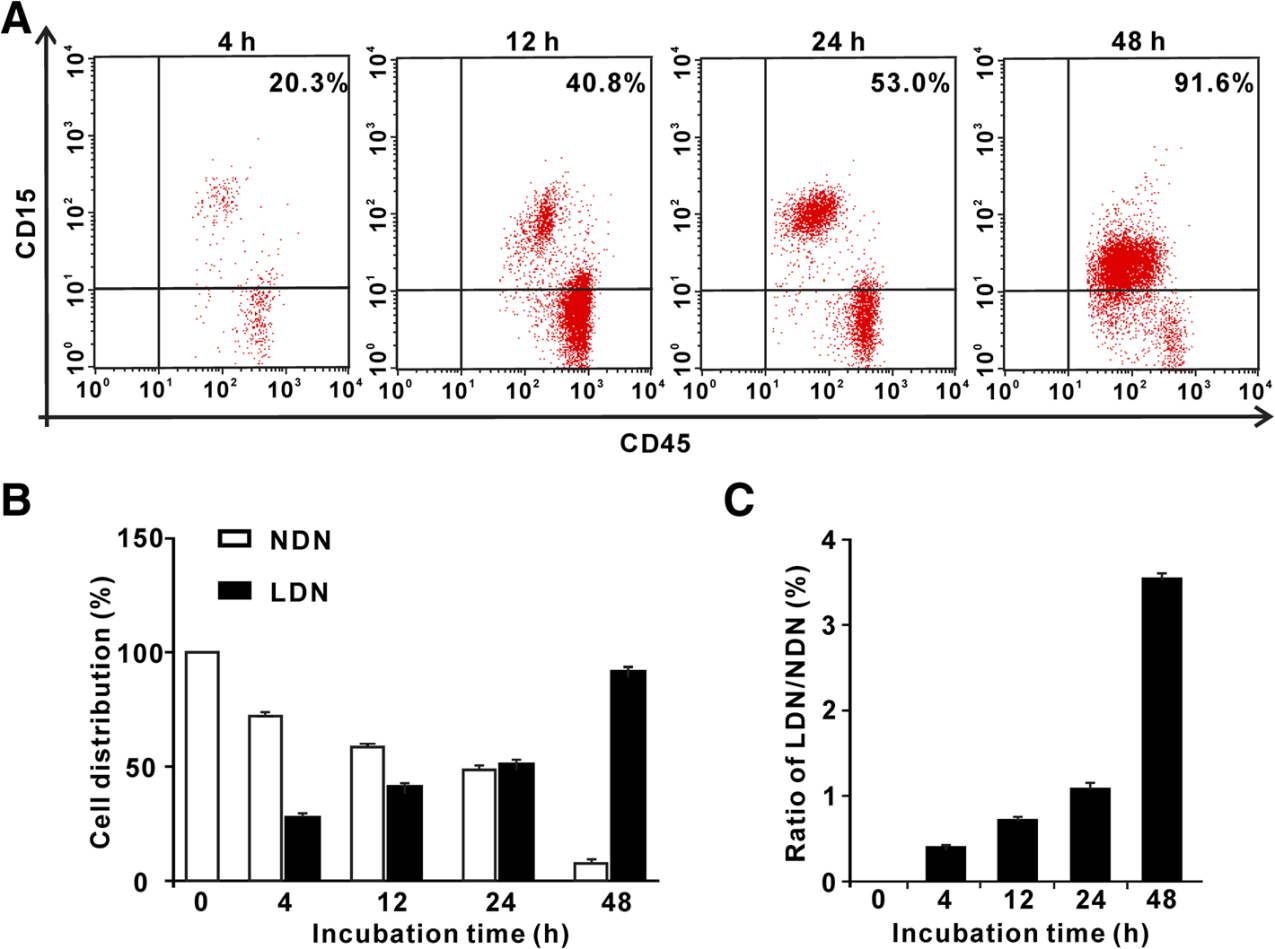
NDNs from normal donors spontaneously switch to LDNs in SFTS condition in vitro. **a** Results showed representative dot plots of NDNs switched to the LDNs. **b** Results showed NDNs switch to the LDNs. **c** The ratio of NDNs switch to the LDNs, suggesting for dynamic transition from NDNs to LDNs. Error bars represent ±SEM

The origin of LDNs in patients of SFTS remain to be characterized. The variation of the microenvironment could obviously affect the conversion of regulatory T cells (Treg cells) to Th cells [15]. The blood from the healthy can generate myeloid-derived suppressor cells (MDSCs), which could be activated by GM-CSF + IL-6, GM-CSF + IL-10, PGE2 [16]. We guessed that similar transitions may occur between NDNs and LDNs. To confirm this hypothesis, we employed highly purified NDNs cells from the healthy donor and incubated NDNs with SFTS patient plasma as described in method. Indeed, we observed that NDNs from the healthy volunteers cultured with the plasma from SFTS patients converted to LDNs (Fig. 2). The percentages of LDNs at different cultured time of 4, 12 and 24 h were 20.3, 40.8 and 53.0%, respectively. The percentages of LDNs continued to increase with time and reached the maximum at 48 h, suggesting that normal NDNs are capable of switching to LDNs under the SFTS condition.

### The SFTSV loads in LDNs were much higher from severe SFTS patients than that from moderate SFTS patients

**Fig. 3:**
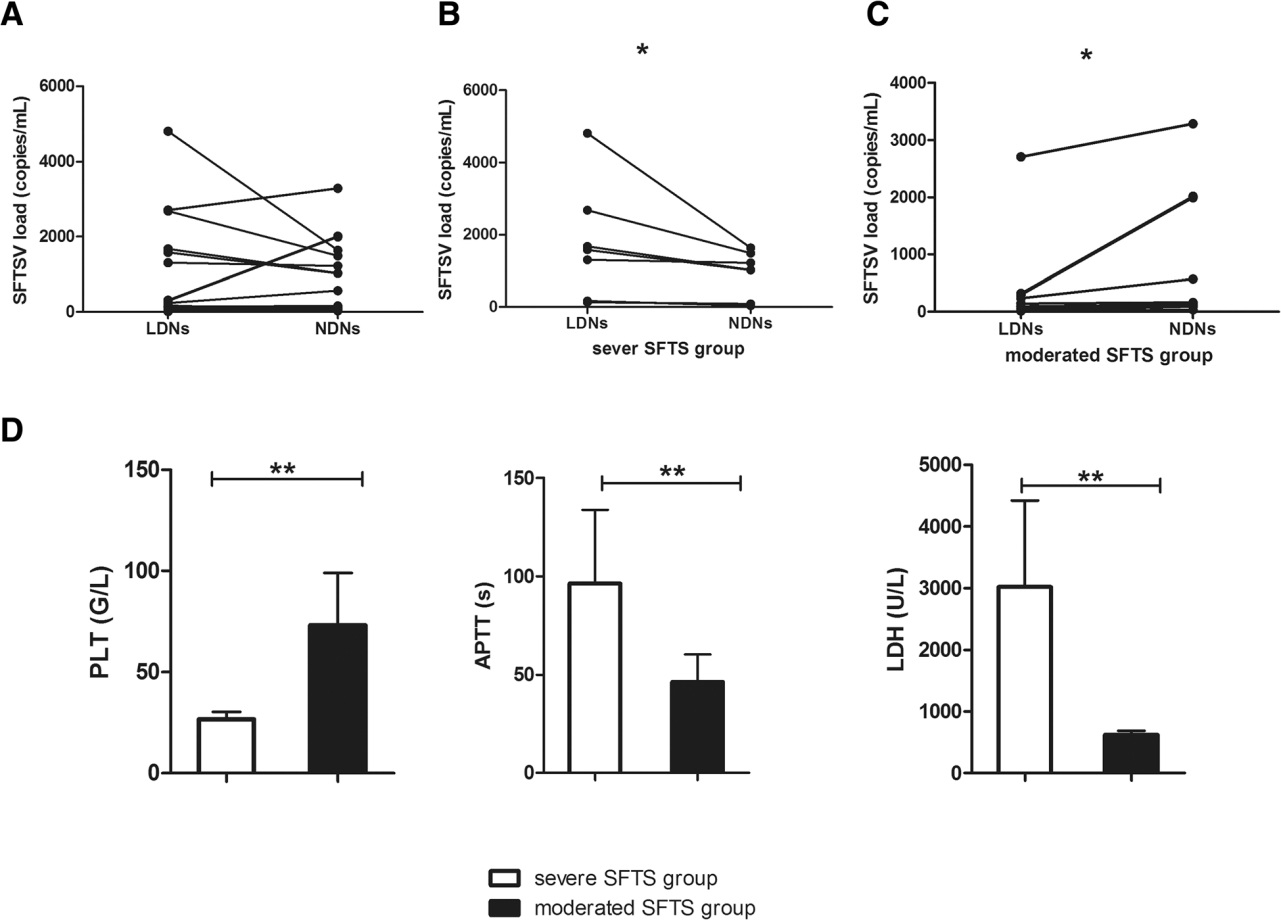
SFTSV loads in LDNs and NDNs from the SFTS patients (*n* = 18). **a** The presence of SFTSV load in LDNs and NDNs from SFTS patients were diversity; **b** The SFTSV load were significantly higher in LDNs than that in NDNs in severe SFTS group; ** *P* < 0.01. **c** The SFTSV load were significantly lower in LDNs than that in NDNs in moderated SFTS patients group. ** *P* < 0.01. **d** The PLT number were significantly lower in severe SFTS group than that in moderated SFTS patients group. ** *P* < 0.01. APTT were significantly longer in severe SFTS group than that in moderated SFTS patients group. ** *P* < 0.01. LDH level were significantly higher in severe SFTS group than that in moderated SFTS patients group.** *P* < 0.01. Error bars represent ±SEM

Neutrophils exhibit potent phagocytic properties via the pathogen annihilation mechanism of their neutrophilic granules. The plethora of performed mediators stored in such neutrophilic granules destroyed the phagocytosed pathogens [17]. Based on this, we evaluated the capability of LDNs in engulfment of SFTSV and explored the SFTSV loads in LDNs and NDNs from the same SFTS patients. As shown in Fig. 3, there was the presence of SFTSV mRNA in LDNs and NDNs from the SFTS patients. The SFTSV loads in LDNs and NDNs from SFTS patients showed diversity (Fig. 3a). Generally, the load of SFTSV was lower in LDNs than that in NDNs of the SFTS patients, but higher than that in serum. We further divided SFTS patients into two groups: severe SFTS patient group and moderate SFTS patient group, according to patient clinical feature, laboratory data and Marshall MODS Score [18]. In severe SFTS patient group, the SFTSV load of LDNs and NDNs were 1768.0286 ± 608.39 and 931.96 ± 239.52 respectively. There was statistical significance between LDNs and NDNs (*p* = 0.018) (Fig. 3b). In moderate SFTS patients group, the SFTSV load were 604.36 ± 315.33 and 1070.91 ± 398.43 in LDNs and NDNs, respectively. There was statistically significant differences between LDNs and NDNs (*p* = 0.038) (Fig. 3c). The SFTSV loads of LDNs were much higher from severe SFTS patients than that from moderate SFTS patients.

Next, we compared the two group patients with blood biochemical indicator, such as platelet number (PLT), activated partial thromboplastin time (APTT) and lactic dehydrogenase (LDH). We found that PLT was 26.71 ± 1.36 in severe SFTS patient group and 73.09 ± 7.81 in moderate SFTS patient group. There were statistically significant differences between the two groups. The APTT in severe SFTS patient group was significantly longer than that in moderate SFTS patient group (96.26 ± 14.15 vs 46.32 ± 4.18, *p* = 0.001). The LDH levels in severe SFTS patient group was 3019.57 ± 528.40 and was 625.82 ± 60.93 in moderate SFTS patient group. There was also statistical significance between two groups (*p* = 0.004) (Fig. 3d).

### LDNs synthesized increased levels of pro-inflammatory cytokines in SFTS patients

**Fig. 4:**
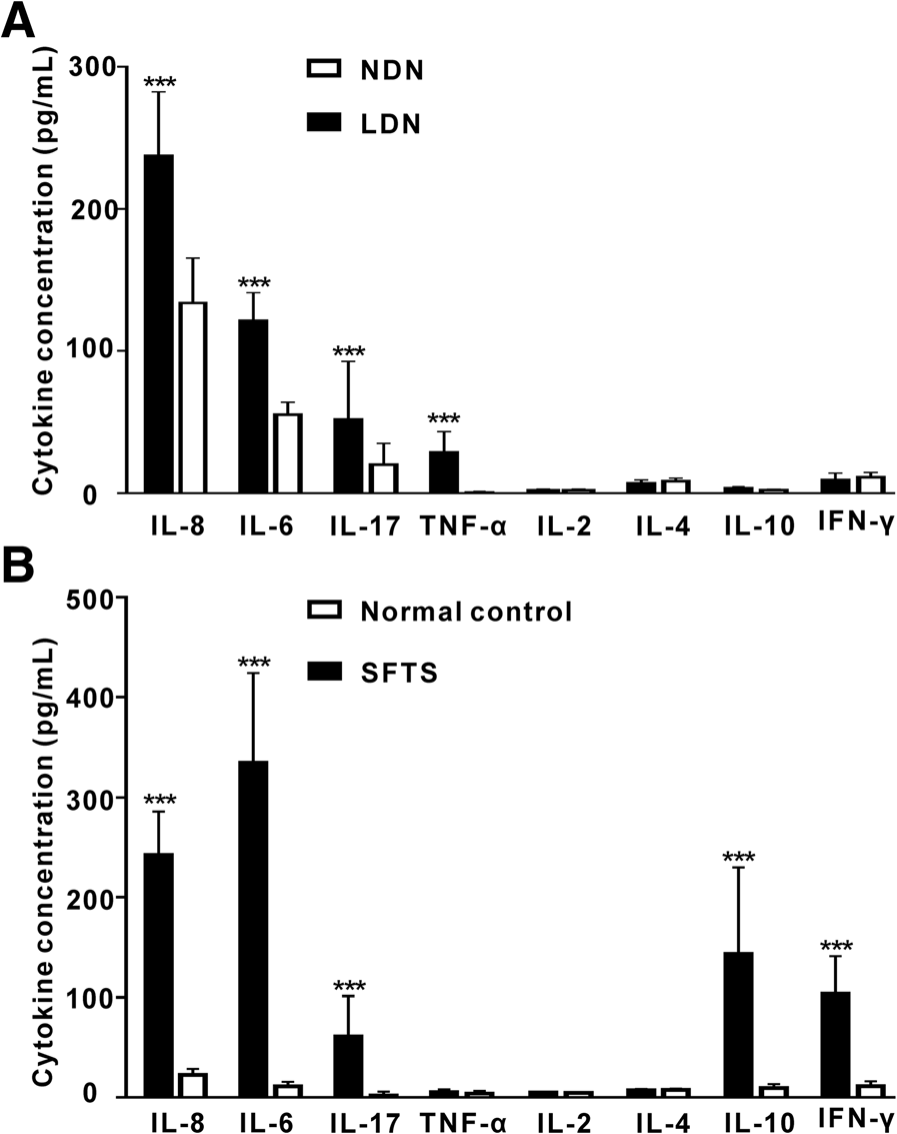
LDNs secrete increased levels of proinflammatory cytokines. **a** Results are shown using stimulated LDNs and measurements were done with supernatants harvested after 48 h in culture; *** *P* < 0.001 when comparing LDNs to NDNs. **b** Bar graphs represent cytokine concentration in SFTS patients serum and normal control (pg/mL). *** *P* < 0.001 when comparing SFTS serum to normal control. Error bars represent ±SD

The neutrophils have a pro-inflammation role, therefore, we performed LDNs and NDNs functional assays to detect the secretion inflammatory cytokines and to validate the secretion function of NDNs and LDNs converted from the NDNs in SFTS micro- environment. The results revealed that IL-6, IL-8, IL-17 and TNF-α level were 121.14 ± 4.47 pg/mL, 237 ± 10.04 pg/mL, 51 ± 9.19 pg/mL and 28.68 ± 3.30, secreted by LDNs, respectively. IL-6, IL-8, IL-17 and TNF-α level were 54.98 ± 1.96 pg/mL, 133.71 ± 7.09 pg/mL, 20.12 ± 3.35 pg/mL, 0.92 ± 0.20 pg/mL, secreted by NDNs, respectively. There were significant differences in IL-6, IL-8, IL-17 and TNF-αsecretion between the LDNs and NDNs, but there were no significant differences in IL-2, IL-4, IL-10 secretion between LDNs and NDNs (Fig. 4a). Figure 4b showed the cytokine concentrations of IL-6, IL-8, IL-17 and TNF-α was significant differences between SFTS patients’ serum and normal control.

### LDNs in SFTS can induce endothelial cell injury

**Fig. 5:**
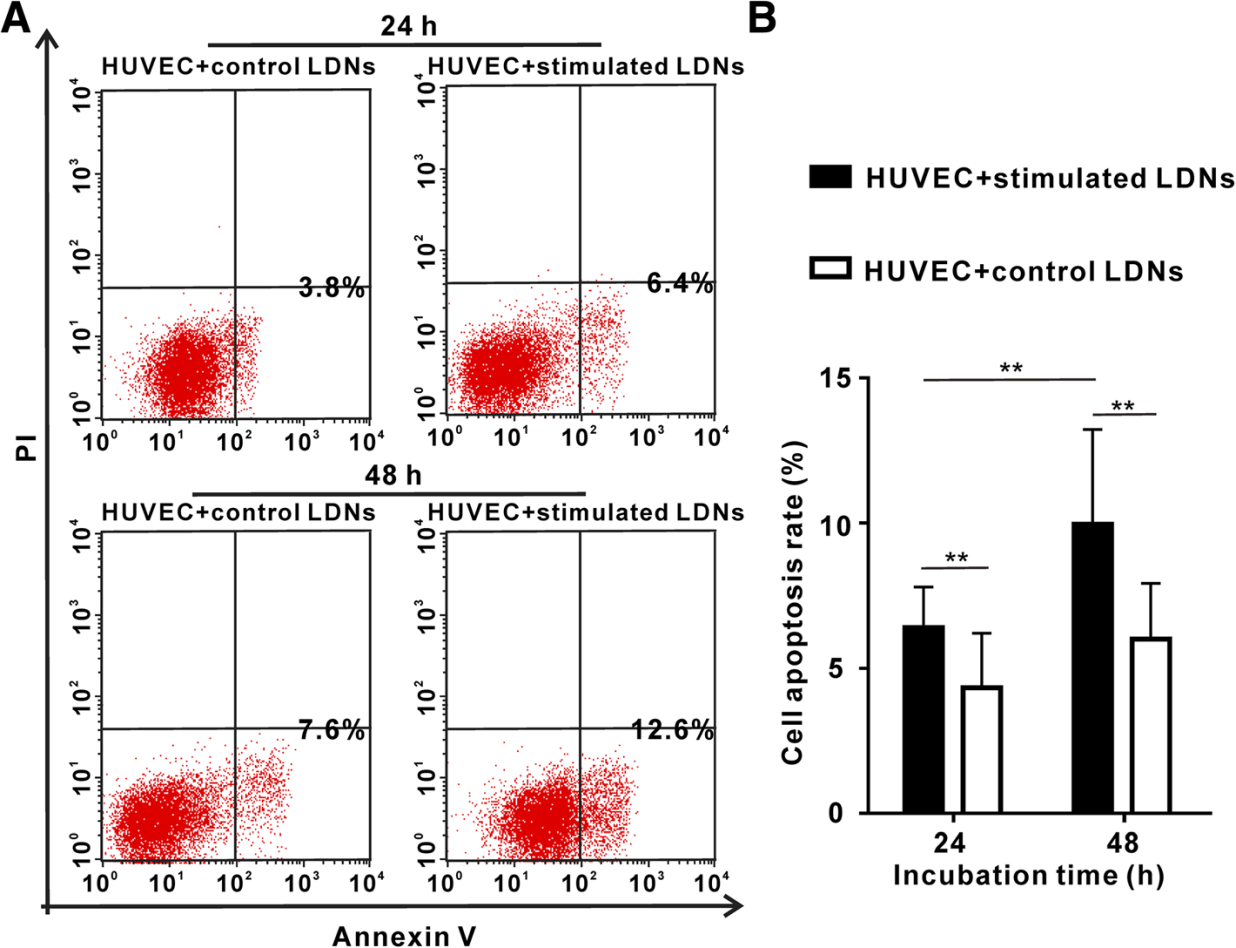
SFTS LDNs are cytotoxic to the endothelium. **a** Results show representative dot plots of endothelial cell apoptosis induced by actived LDNs or control neutrophils. **b** Bar graphs represent % of apoptotic HUVECs after 24 h or 48 h exposure to LDNs or control neutrophils. **p < 0.01 when comparing actived LDNs to normal control. Error bars represent ±SEM

In vitro, neutrophil-isolated defensins can be cytotoxic to epithelial cells, for clearing virally infected cells but also causing tissue injury during severe viral infections [19]. Given the potential role of neutrophils in the induction of damages to the endothelium in other pathologic conditions [20]. We examined whether LDNs can harm endothelial cells. Our results revealed that SFTS LDNs induced significantly higher levels of cytotoxicity of endothelial cells than control neutrophils (Fig. 5). The apoptosis rate of HUVEC cells was markedly increased after co-cultured with SFTS LDNs via transwell which could eliminate direct contact between the LDNs and the endothelial cells.

## Discussion

In our current work, we further demonstrated that circulating neutrophils in SFTS were presence of distinct subpopulations, LDNs and NDNs. SFTSV infections resulted in high proportion of LDNs in PBMCs. The proportion of LDNs not only elevated in mycobacterial infection [11], but also increased in circulating blood of patients with viral infection [13], cancer [12]. Our results showed that the percentage of LDNs was (46.80 ± 14.1)% in circulating blood SFTS patients, increasing dramatically to the extent that LDNs often became the dominant circulating neutrophil population with The LDNs may play more important role in SFTS disease.

From prior studies, neutrophils are formed within the bone marrow during hematopoiesis in response to several cytokines, principally granulocyte colony–stimulating factor [21]. Denny MF, et al. reported that the LDNs do not seem to represent a population of in vivo activated and degranulated neutrophils [10]. We employed highly purified populations of the NDNs cells from the healthy donor and tested whether the transition between NDNs and LDNs can be mimicked in SFTS conditions, ex vivo. As expected, NDNs from the healthy donor could convert to LDNs under cultured with SFTS patients’ plasma, suggesting that the NDNs retained the developmental plasticity and could convert to LDNs which was one of the source increased percentage of LDNs in SFTS.

Neutrophils have the capacity to respond to a wide range of stimuli. There was not only presence of increased pro-inflammation cytokines, but also presence of SFTSV in SFTS patients’ plasma. At this moment, it is unclear which regulates the transition from NDNs to LDNs. Although we are making SFTS animal models, but the difficulty in handling SFTS animal model has hampered our study in vivo for confirming the experiments on transition NDNs to LDNs. Anyway, our results challenge the current concept of circulating neutrophils as a fully differentiated homogenous cell population that has limited plasticity.

We demonstrated that SFTSV load in LDNs and NDNs were diversified. From a global perspective, the load of SFTSV was lower in LDNs than that in NDNs of the SFTS patients. But careful analysis showed that the SFTSV loads of LDNs were much higher from severe SFTS patients than that from moderate SFTS patients.

Although the SFTSV were in LDNs, it was not clear whether this was due to uptake of the virus itself or by active infection and propagation of the virus within the LDNs. The neutrophils can actively take up IAV in vitro [22]. One study showed that LDNs had the function of phagocytose and could phagocytose fewer FITC-labeled microbeads [12]. Based on those studies, we inferred that LDNs in SFTS could engulf SFTSV and disseminated the viruses.

In this study we found that platelet number was lower and the APTT was longer, and the LDH level was higher when SFTSV load were high in LDNs. This phenomenon may be a contributing factor of bleeding by patients with SFTS, but the specific mechanism is not fully understood. In our study, 60% of SFTS patients were in moderate patient group, and 40% of SFTS patients were in severe patient group. That is why generally the load of SFTSV was lower in LDNs than that in NDNs of the SFTS patients. In clinical trials, almost all the SFTS patients were curable in moderate patient group, but almost all the death patients were in the severe patient group. Together, those findings suggested that high SFTSV load in LDNs was an indicator of SFTS disease severity and prognosis.

Controversy surrounds the neutrophil function in viral infection because neutrophils in viral infections were shown to have both beneficial and harmful effects [23, 24]. On the one hand, the neutrophils exhibit a strong ability to mediate direct antiviral effects after viral infection. They can rapidly initiate an antiviral program and contributes to its clearance [25]. On the other hand, neutrophils have also been proposed to act as “Trojan horses” for the dissemination of virus, acting as a vehicle for viral replication, proliferation [26, 27]. LDNs from severe SFTS patients can engulf more SFTSV. It is not known whether LDNs from SFTS patients take up viral particles and kill them or act as a vehicle for viral replication, proliferation. Nonetheless, further investigations are needed to elucidate the functional role of LDNs in SFTSV infection.

The percentages of LDNs are not only elevated in bacterial infection patients PBMCs [11], but also increased in viral infection patients PBMCs [13]. LDNs may have special functional role in viral infection. Generally, SFTSV infection induces a cytokine storm in SFTS patients [28]. Our results showed that LDNs secreted more IL-6, IL-8, IL-17 and TNF-α levels than that of NDNs. IL-6, IL-8, TNF-α are proinflammatory cytokines, indicating that the LDNs secreted higher proinflammatory cytokines than NDNs upon SFTSV infection, and these might be one of the sources of proinflammatory cytokines in SFTS.

Our results diaplayed that the active LDNs infected by SFTSV could induce more endothelial cytotoxic when compared to control LDNs. This observation that LDNs from SFTS were cytotoxic to the endothelium suggested that they might play an important role in the induction of vascular damage in SFTS. LDNs were active and could synthesize more cytokines. Based on those, we inferred that cytokines secreted by active-LDNs might be resulted in the violent immune reaction and resulting in vascular damage. The neutrophil function in virus infection exhibit both protective and pathological functions [8]. Based on our findings as mentioned above, we reasoned that NDNs might play a protective role, whereas the LDNs may associate with proinflammation activity in SFTSV infection.

## Conclusions

In conclusion, we demonstrated that the percentage of LDNs was significantly higher in SFTS patients. More importantly, the NDNs retained developmental plasticity and could convert to LDNs in SFTS environment. The LDNs in severe SFTS patient could engulf more SFTSV. In addition, LDNs could synthesize increased levels of pro-inflammatory cytokines and induce endothelial cell injury. Therefore, the LDNs may exhibit pro-inflammation functions in SFTSV infection.

## Abbreviations

APTT: Activated partial thromboplastin time
HUVEC: Human umbilical vein endothelial cell
IFN: Interferon
IL: Interleukin
LDH: Lactic dehydrogenase
LDNs: Low-density neutrophils
NDNs: Normal-density neutrophils
PLT: Platele
RBC: Red blood cell
SFTS: Severe fever with thrombocytopenia syndrome
SFTSV: Severe fever with thrombocytopenia syndrome virus
TNF: Tumor necrosis factor

## References

[1] Yu XJ, Liang MF, Zhang SY (2011). Fever with thrombocytopenia associated with a novel bunyavirus in China. N Engl J Med.

[2] Kim WY, Choi W, Park SW (2015). Nosocomial transmission of severe fever with thrombocytopenia syndrome in Korea. Clin Infect Dis.

[3] Yoshikawa T, Shimojima M, Fukushi S (2015). Phylogenetic and geographic relationships of severe fever with thrombocytopenia syndrome virus in China, South Korea, and Japan. J Infect Dis.

[4] Zhan JB, Wang Q, Cheng J (2017). Current status of severe fever with thrombocytopenia syndrome in China. Virol Sin.

[5] Sun L, Hu Y, Niyonsaba A (2014). Detection and evaluation of immunofunction of patients with severe fever with thrombocytopenia syndrome. Clin Exp Med.

[6] Li J, Han Y, Xing Y (2014). Concurrent measurement of dynamic changes in viral load, serum enzymes, T cell subsets, and cytokines in patients with severe fever with thrombocytopenia syndrome. PLoS One.

[7] Ding YP, Liang MF, Ye JB (2014). Prognostic value of clinical and immunological markers in acute phase of SFTS virus infection. Clin Microbiol Infect.

[8] Galani IE, Andreakos E (2015). Neutrophils in viral infections: current concepts and caveats. J Leukoc Biol.

[9] Tsuda Y, Takahashi H, Kobayashi M (2004). Three different neutrophil subsets exhibited in mice with different susceptibilities to infection by methicillin-resistant Staphylococcus aureus. Immunity.

[10] Denny MF, Yalavarthi S, Zhao W (2010). A distinct subset of proinflammatory neutrophils isolated from patients with systemic lupus erythematosus induces vascular damage and synthesizes type I IFNs. J Immunol.

[11] Deng Y, Ye J, Luo Q (2016). Low-density granulocytes are elevated in mycobacterial infection and associated with the severity of tuberculosis. PLoS One.

[12] Sagiv JY, Michaeli J, Assi S (2015). Phenotypic diversity and plasticity in circulating neutrophil subpopulations in cancer. Cell Rep.

[13] Cloke T, Munder M, Taylor G (2012). Characterization of a novel population of low-density granulocytes associated with disease severity in HIV-1 infection. PLoS One.

[14] Li YJ, Peng C, Li HY (2017). Low-density neutrophils in severe fever with thrombocytopenia syndrome (SFTS) display decreased function to phagocytose SFTS virus and enhanced capacity to synthesize cytokines. Int J Clin Exp Pathol.

[15] Yang XO, Nurieva R, Martinez GJ (2008). Molecular antagonism and plasticity of regulatory and inflammatory T cell programs. Immunity.

[16] Greten TF, Manns MP, Korangy F (2011). Myeloid derived suppressor cells in human diseases. Int Immunopharmacol.

[17] Dale DC, Boxer L, Liles WC (2008). The phagocytes: neutrophilsand monocytes. Blood.

[18] Jie SH, Zhou Y, Sun LP (2013). Close correlation between development of MODS during the initial 72h of hospitalization and hospital mortality in severe fever with thrombocytopenia syndrome. J Huazhong Univ Sci Technol.

[19] Okrent DG, Lichtenstein AK, Ganz T (1990). Direct cytotoxicity ofpolymorphonuclear leukocyte granule proteins to human lung-derivedcells and endothelial cells. Am Rev Respir Dis.

[20] Ward PA, Varani J (1990). Mechanisms of neutrophil-mediated killing of endothelial cells. J Leukoc Biol.

[21] Borregaard N (2010). Neutrophils from marrow to microbes. Immunity.

[22] Ratcliffe D, Migliorisi G, Cramer E (1992). Translocation of influenzavirus by migrating neutrophils. Cell Mol Biol.

[23] Zhou J, Stohlman SA, Hinton DR (2003). Neutrophils promote mononuclear cell infiltration during viral-induced encephalitis. J Immunol.

[24] Tumpey TM, Chen SH, Oakes JE (1996). Neutrophil-mediated suppression of virus replication after herpes simplex virus type 1 infection of the murine cornea. J Virol.

[25] Tamassia N, Le Moigne V, Rossato M (2008). Activation of an immunoregulatory and antiviral gene expressionprogram in poly(I:C)-transfected human neutrophils. J Immunol.

[26] Zhao Y, Lu M, Lau LT (2008). Neutrophils may be a vehicle for viralreplication and dissemination in human H5N1 avian influenza. Clin Infect Dis.

[27] Bai F, Kong KF, Dai J (2010). A paradoxical role for neutrophils in the pathogenesis of West Nile virus. J Infect Dis.

[28] Sun Y, Jin C, Zhan F (2012). Host cytokine storm is associated with disease severity of severe fever with thrombocytopenia syndrome. J Infect Dis.

